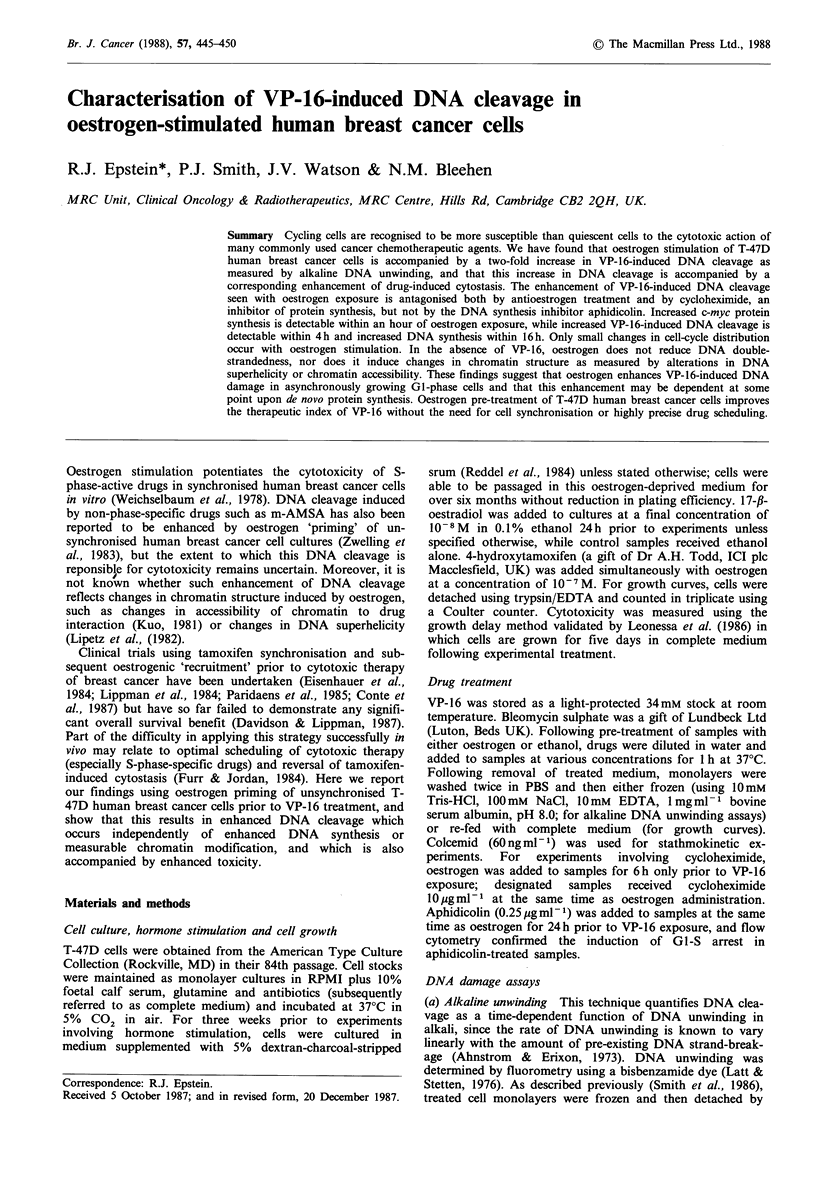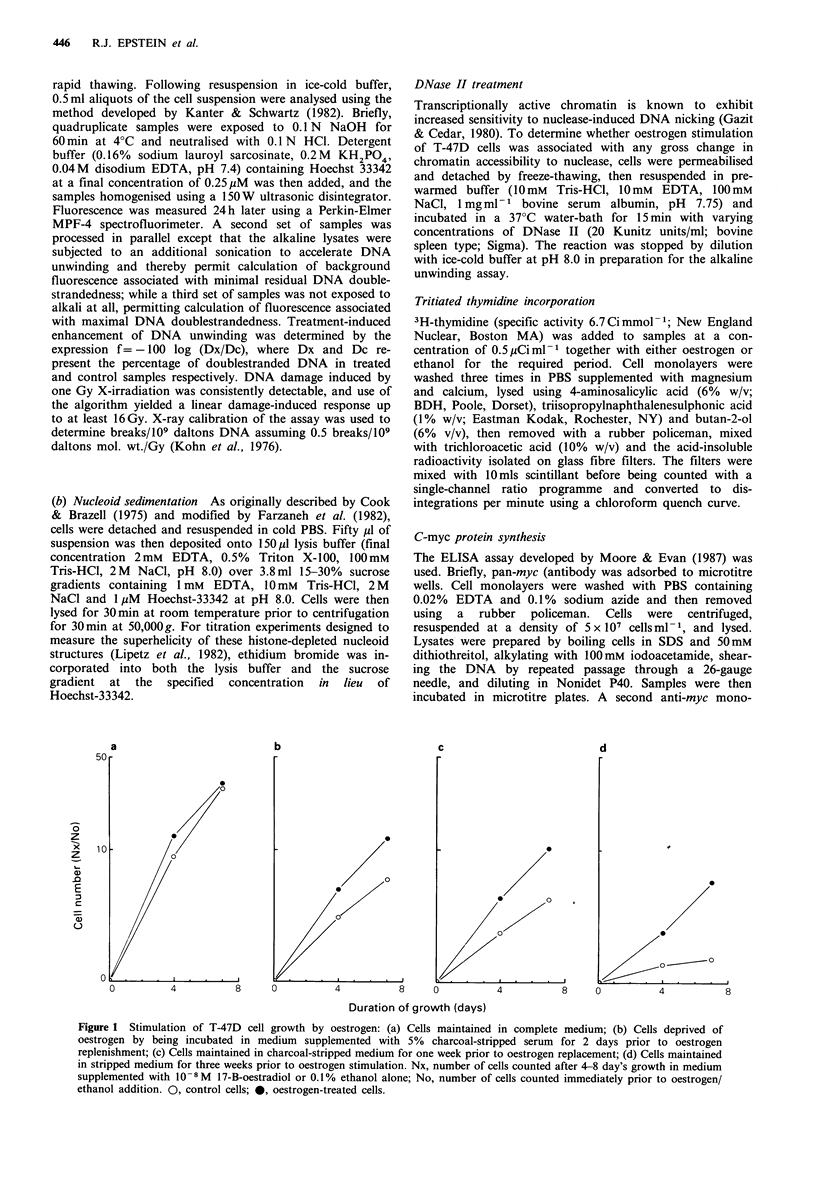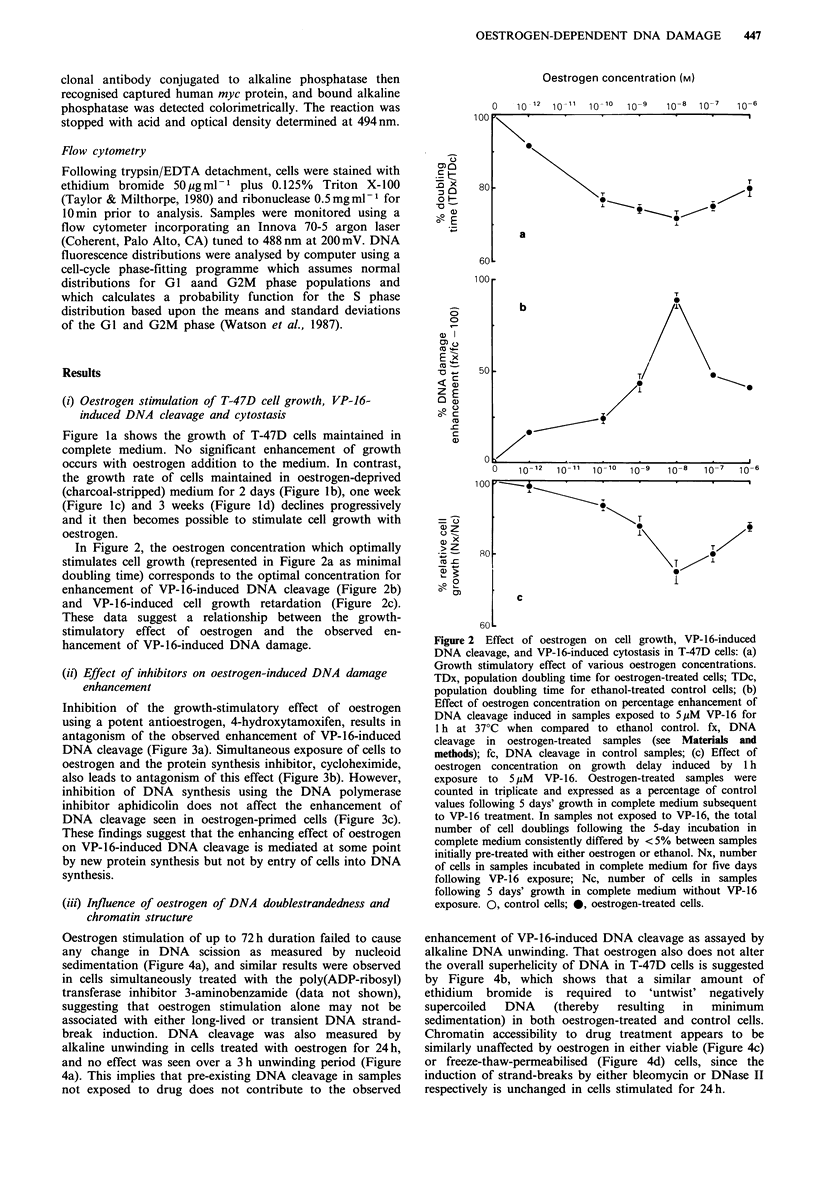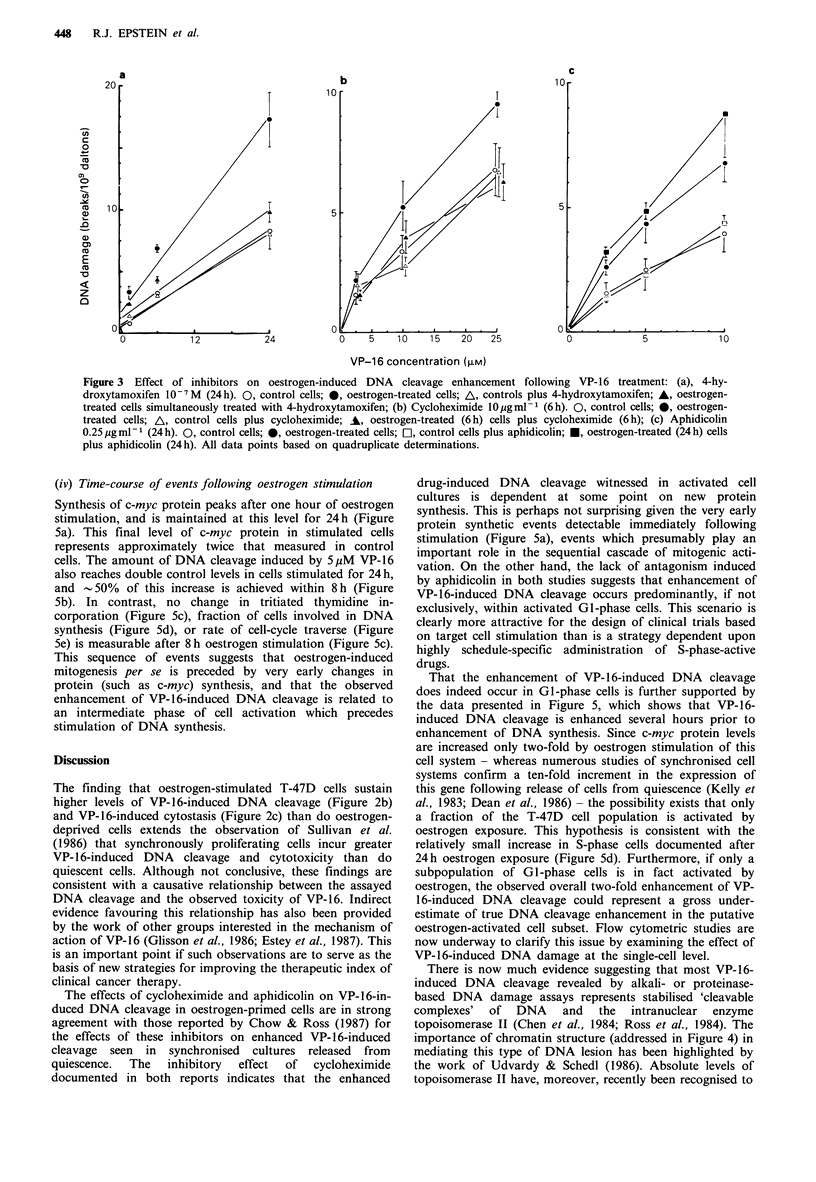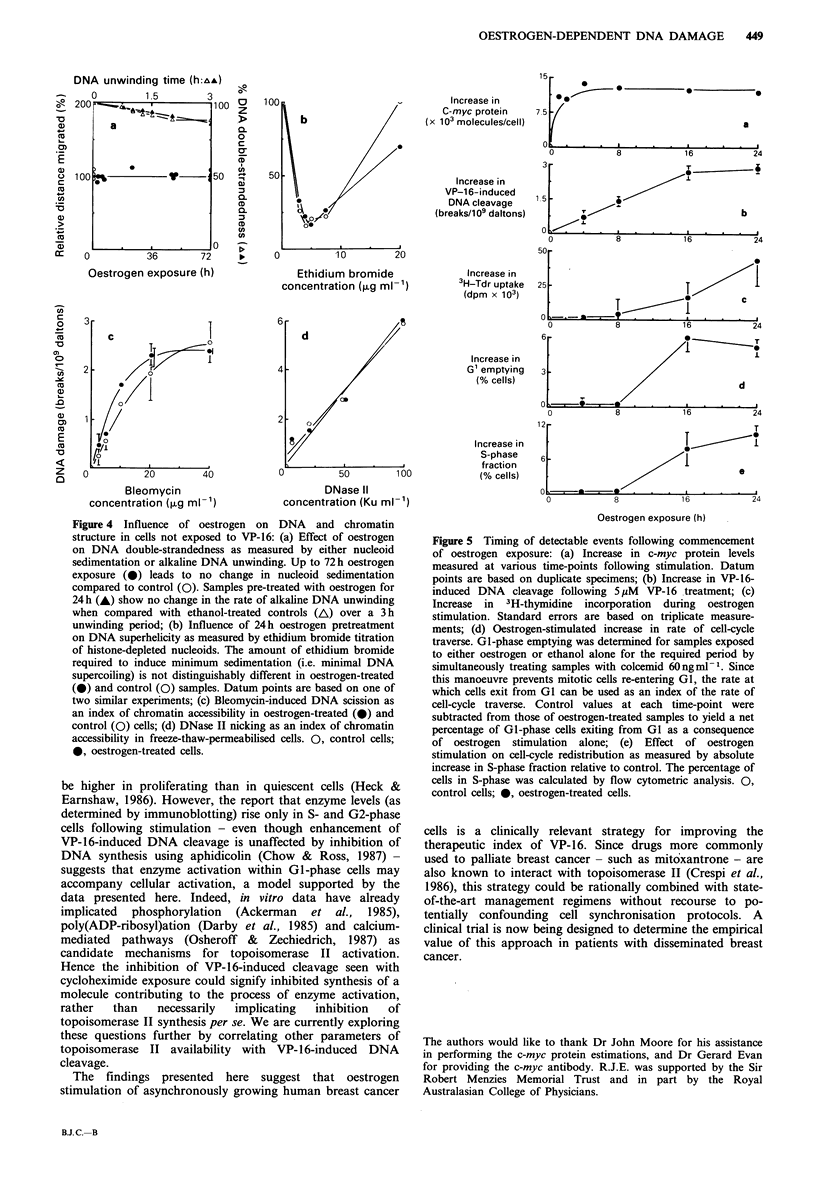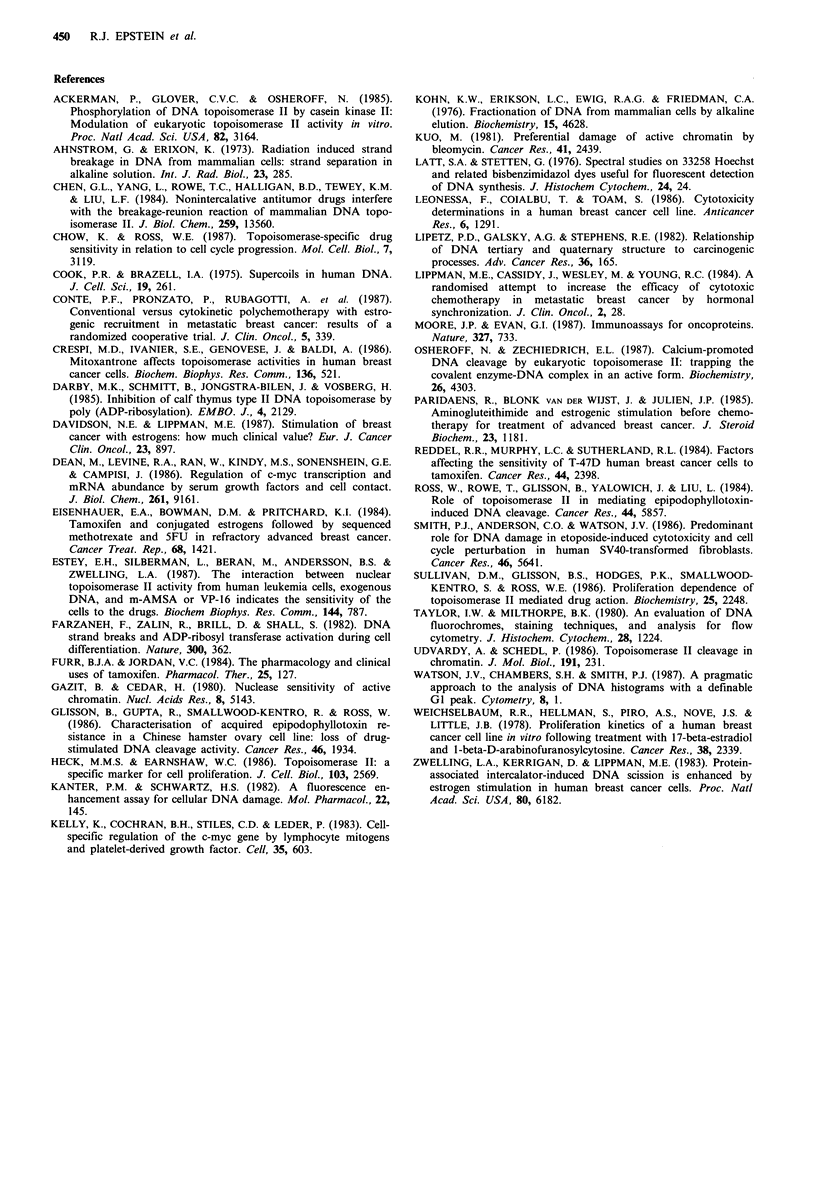# Characterisation of VP-16-induced DNA cleavage in oestrogen-stimulated human breast cancer cells.

**DOI:** 10.1038/bjc.1988.104

**Published:** 1988-05

**Authors:** R. J. Epstein, P. J. Smith, J. V. Watson, N. M. Bleehen

**Affiliations:** MRC Unit, MRC Centre, Cambridge, UK.

## Abstract

Cycling cells are recognised to be more susceptible than quiescent cells to the cytotoxic action of many commonly used cancer chemotherapeutic agents. We have found that oestrogen stimulation of T-47D human breast cancer cells is accompanied by a two-fold increase in VP-16-induced DNA cleavage as measured by alkaline DNA unwinding, and that this increase in DNA cleavage is accompanied by a corresponding enhancement of drug-induced cytostasis. The enhancement of VP-16-induced DNA cleavage seen with oestrogen exposure is antagonised both by antioestrogen treatment and by cycloheximide, an inhibitor of protein synthesis, but not by the DNA synthesis inhibitor aphidicolin. Increased c-myc protein synthesis is detectable within an hour of oestrogen exposure, while increased VP-16-induced DNA cleavage is detectable within 4h and increased DNA synthesis within 16h. Only small changes in cell-cycle distribution occur with oestrogen stimulation. In the absence of VP-16, oestrogen does not reduce DNA double-strandedness, nor does it induce changes in chromatin structure as measured by alterations in DNA superhelicity or chromatin accessibility. These findings suggest that oestrogen enhances VP-16-induced DNA damage in asynchronously growing G1-phase cells and that this enhancement may be dependent at some point upon de novo protein synthesis. Oestrogen pre-treatment of T-47D human breast cancer cells improves the therapeutic index of VP-16 without the need for cell synchronisation or highly precise drug scheduling.


*Br. J. Cancer* (1988), **57**, 445–450 © The Macmillan Press Ltd., 1988

### Characterisation of VP-16-induced DNA cleavage in oestrogen-stimulated human breast cancer cells

R.J. Epstein*, P.J. Smith, J.V. Watson & N.M. Bleehen

*MRC Unit, Clinical Oncology & Radiotherapeutics, MRC Centre, Hills Rd, Cambridge CB2 2QH, UK.*

**Summary** Cycling cells are recognised to be more susceptible than quiescent cells to the cytotoxic action of many commonly used cancer chemotherapeutic agents. We have found that oestrogen stimulation of T-47D human breast cancer cells is accompanied by a two-fold increase in VP-16-induced DNA cleavage as measured by alkaline DNA unwinding, and that this increase in DNA cleavage is accompanied by a corresponding enhancement of drug-induced cytostasis. The enhancement of VP-16-induced DNA cleavage seen with oestrogen exposure is antagonised both by antioestrogen treatment and by cycloheximide, an inhibitor of protein synthesis, but not by the DNA synthesis inhibitor aphidicolin. Increased c-*myc* protein synthesis is detectable within an hour of oestrogen exposure, while increased VP-16-induced DNA cleavage is detectable within 4 h and increased DNA synthesis within 16 h. Only small changes in cell-cycle distribution occur with oestrogen stimulation. In the absence of VP-16, oestrogen does not reduce DNA double-strandedness, nor does it induce changes in chromatin structure as measured by alterations in DNA superhelicity or chromatin accessibility. These findings suggest that oestrogen enhances VP-16-induced DNA damage in asynchronously growing G1-phase cells and that this enhancement may be dependent at some point upon *de novo* protein synthesis. Oestrogen pre-treatment of T-47D human breast cancer cells improves the therapeutic index of VP-16 without the need for cell synchronisation or highly precise drug scheduling.

Oestrogen stimulation potentiates the cytotoxicity of S-phase-active drugs in synchronised human breast cancer cells *in vitro* (Weichselbaum *et al.*, 1978). DNA cleavage induced by non-phase-specific drugs such as m-AMSA has also been reported to be enhanced by oestrogen 'priming' of unsynchronised human breast cancer cell cultures (Zwelling *et al.*, 1983), but the extent to which this DNA cleavage is reponsible for cytotoxicity remains uncertain. Moreover, it is not known whether such enhancement of DNA cleavage reflects changes in chromatin structure induced by oestrogen, such as changes in accessibility of chromatin to drug interaction (Kuo, 1981) or changes in DNA superhelicity (Lipetz *et al.*, (1982).

Clinical trials using tamoxifen synchronisation and subsequent oestrogenic 'recruitment' prior to cytotoxic therapy of breast cancer have been undertaken (Eisenhauer *et al.*, 1984; Lippman *et al.*, 1984; Paridaens *et al.*, 1985; Conte *et al.*, 1987) but have so far failed to demonstrate any significant overall survival benefit (Davidson & Lippman, 1987). Part of the difficulty in applying this strategy successfully *in vivo* may relate to optimal scheduling of cytotoxic therapy (especially S-phase-specific drugs) and reversal of tamoxifen-induced cytostasis (Furr & Jordan, 1984). Here we report our findings using oestrogen priming of unsynchronised T-47D human breast cancer cells prior to VP-16 treatment, and show that this results in enhanced DNA cleavage which occurs independently of enhanced DNA synthesis or measurable chromatin modification, and which is also accompanied by enhanced toxicity.

### Materials and methods

### *Cell culture, hormone stimulation and cell growth*

T-47D cells were obtained from the American Type Culture Collection (Rockville, MD) in their 84th passage. Cell stocks were maintained as monolayer cultures in RPMI plus 10% foetal calf serum, glutamine and antibiotics (subsequently referred to as complete medium) and incubated at 37°C in 5% $CO_2$ in air. For three weeks prior to experiments involving hormone stimulation, cells were cultured in medium supplemented with 5% dextran-charcoal-stripped srum (Reddel *et al.*, 1984) unless stated otherwise; cells were able to be passaged in this oestrogen-deprived medium for over six months without reduction in plating efficiency. 17-$\beta$-oestradiol was added to cultures at a final concentration of $10^{-8}$ M in 0.1% ethanol 24 h prior to experiments unless specified otherwise, while control samples received ethanol alone. 4-hydroxytamoxifen (a gift of Dr A.H. Todd, ICI plc Macclesfield, UK) was added simultaneously with oestrogen at a concentration of $10^{-7}$ M. For growth curves, cells were detached using trypsin/EDTA and counted in triplicate using a Coulter counter. Cytotoxicity was measured using the growth delay method validated by Leonessa *et al.* (1986) in which cells are grown for five days in complete medium following experimental treatment.

### *Drug treatment*

VP-16 was stored as a light-protected 34 mM stock at room temperature. Bleomycin sulphate was a gift of Lundbeck Ltd (Luton, Beds UK). Following pre-treatment of samples with either oestrogen or ethanol, drugs were diluted in water and added to samples at various concentrations for 1 h at 37°C. Following removal of treated medium, monolayers were washed twice in PBS and then either frozen (using 10 mM Tris-HCl, 100 mM NaCl, 10 mM EDTA, 1 mg ml$^{-1}$ bovine serum albumin, pH 8.0; for alkaline DNA unwinding assays) or re-fed with complete medium (for growth curves). Colcemid (60 ng ml$^{-1}$) was used for stathmokinetic experiments. For experiments involving cycloheximide, oestrogen was added to samples for 6 h only prior to VP-16 exposure; designated samples received cycloheximide 10 $\mu$g ml$^{-1}$ at the same time as oestrogen administration. Aphidicolin (0.25 $\mu$g ml$^{-1}$) was added to samples at the same time as oestrogen for 24 h prior to VP-16 exposure, and flow cytometry confirmed the induction of G1-S arrest in aphidicolin-treated samples.

### *DNA damage assays*

(*a*) *Alkaline unwinding* This technique quantifies DNA cleavage as a time-dependent function of DNA unwinding in alkali, since the rate of DNA unwinding is known to vary linearly with the amount of pre-existing DNA strand-breakage (Ahnstrom & Erixon, 1973). DNA unwinding was determined by fluorometry using a bisbenzamide dye (Latt & Stetten, 1976). As described previously (Smith *et al.*, 1986), treated cell monolayers were frozen and then detached by

Correspondence: R.J. Epstein.
Received 5 October 1987; and in revised form, 20 December 1987.

rapid thawing. Following resuspension in ice-cold buffer, 0.5 ml aliquots of the cell suspension were analysed using the method developed by Kanter & Schwartz (1982). Briefly, quadruplicate samples were exposed to 0.1 N NaOH for 60 min at 4°C and neutralised with 0.1 N HCl. Detergent buffer (0.16% sodium lauroyl sarcosinate, 0.2 M $KH_2PO_4$, 0.04 M disodium EDTA, pH 7.4) containing Hoechst 33342 at a final concentration of 0.25 $\mu$M was then added, and the samples homogenised using a 150 W ultrasonic disintegrator. Fluorescence was measured 24 h later using a Perkin-Elmer MPF-4 spectrofluorimeter. A second set of samples was processed in parallel except that the alkaline lysates were subjected to an additional sonication to accelerate DNA unwinding and thereby permit calculation of background fluorescence associated with minimal residual DNA double-strandedness; while a third set of samples was not exposed to alkali at all, permitting calculation of fluorescence associated with maximal DNA doublestrandedness. Treatment-induced enhancement of DNA unwinding was determined by the expression $f = -100 \log (Dx/Dc)$, where Dx and Dc represent the percentage of doublestranded DNA in treated and control samples respectively. DNA damage induced by one Gy X-irradiation was consistently detectable, and use of the algorithm yielded a linear damage-induced response up to at least 16 Gy. X-ray calibration of the assay was used to determine breaks/$10^9$ daltons DNA assuming 0.5 breaks/$10^9$ daltons mol. wt./Gy (Kohn *et al.*, 1976).

(*b*) *Nucleoid sedimentation* As originally described by Cook & Brazell (1975) and modified by Farzaneh *et al.* (1982), cells were detached and resuspended in cold PBS. Fifty $\mu$l of suspension was then deposited onto 150 $\mu$l lysis buffer (final concentration 2 mM EDTA, 0.5% Triton X-100, 100 mM Tris-HCl, 2 M NaCl, pH 8.0) over 3.8 ml 15–30% sucrose gradients containing 1 mM EDTA, 10 mM Tris-HCl, 2 M NaCl and 1 $\mu$M Hoechst-33342 at pH 8.0. Cells were then lysed for 30 min at room temperature prior to centrifugation for 30 min at 50,000 *g*. For titration experiments designed to measure the superhelicity of these histone-depleted nucleoid structures (Lipetz *et al.*, 1982), ethidium bromide was incorporated into both the lysis buffer and the sucrose gradient at the specified concentration *in lieu* of Hoechst-33342.

*DNase II treatment*

Transcriptionally active chromatin is known to exhibit increased sensitivity to nuclease-induced DNA nicking (Gazit & Cedar, 1980). To determine whether oestrogen stimulation of T-47D cells was associated with any gross change in chromatin accessibility to nuclease, cells were permeabilised and detached by freeze-thawing, then resuspended in pre-warmed buffer (10 mM Tris-HCl, 10 mM EDTA, 100 mM NaCl, 1 mg ml$^{-1}$ bovine serum albumin, pH 7.75) and incubated in a 37°C water-bath for 15 min with varying concentrations of DNase II (20 Kunitz units/ml; bovine spleen type; Sigma). The reaction was stopped by dilution with ice-cold buffer at pH 8.0 in preparation for the alkaline unwinding assay.

*Tritiated thymidine incorporation*

$^3$H-thymidine (specific activity 6.7 Ci mmol$^{-1}$; New England Nuclear, Boston MA) was added to samples at a concentration of 0.5 $\mu$Ci ml$^{-1}$ together with either oestrogen or ethanol for the required period. Cell monolayers were washed three times in PBS supplemented with magnesium and calcium, lysed using 4-aminosalicylic acid (6% w/v; BDH, Poole, Dorset), triisopropylnaphthalenesulphonic acid (1% w/v; Eastman Kodak, Rochester, NY) and butan-2-ol (6% v/v), then removed with a rubber policeman, mixed with trichloroacetic acid (10% w/v) and the acid-insoluble radioactivity isolated on glass fibre filters. The filters were mixed with 10 mls scintillant before being counted with a single-channel ratio programme and converted to disintegrations per minute using a chloroform quench curve.

*C-*myc *protein synthesis*

The ELISA assay developed by Moore & Evan (1987) was used. Briefly, pan-*myc* (antibody was adsorbed to microtitre wells. Cell monolayers were washed with PBS containing 0.02% EDTA and 0.1% sodium azide and then removed using a rubber policeman. Cells were centrifuged, resuspended at a density of $5 \times 10^7$ cells ml$^{-1}$, and lysed. Lysates were prepared by boiling cells in SDS and 50 mM dithiothreitol, alkylating with 100 mM iodoacetamide, shearing the DNA by repeated passage through a 26-gauge needle, and diluting in Nonidet P40. Samples were then incubated in microtitre plates. A second anti-*myc* mono-

**Figure 1** Stimulation of T-47D cell growth by oestrogen: (a) Cells maintained in complete medium; (b) Cells deprived of oestrogen by being incubated in medium supplemented with 5% charcoal-stripped serum for 2 days prior to oestrogen replenishment; (c) Cells maintained in charcoal-stripped medium for one week prior to oestrogen replacement; (d) Cells maintained in stripped medium for three weeks prior to oestrogen stimulation. Nx, number of cells counted after 4–8 day's growth in medium supplemented with $10^{-8}$ M 17-B-oestradiol or 0.1% ethanol alone; No, number of cells counted immediately prior to oestrogen/ethanol addition. ○, control cells; ●, oestrogen-treated cells.

clonal antibody conjugated to alkaline phosphatase then recognised captured human *myc* protein, and bound alkaline phosphatase was detected colorimetrically. The reaction was stopped with acid and optical density determined at 494 nm.

*Flow cytometry*

Following trypsin/EDTA detachment, cells were stained with ethidium bromide 50 $\mu$g ml$^{-1}$ plus 0.125% Triton X-100 (Taylor & Milthorpe, 1980) and ribonuclease 0.5 mg ml$^{-1}$ for 10 min prior to analysis. Samples were monitored using a flow cytometer incorporating an Innova 70-5 argon laser (Coherent, Palo Alto, CA) tuned to 488 nm at 200 mV. DNA fluorescence distributions were analysed by computer using a cell-cycle phase-fitting programme which assumes normal distributions for G1 aand G2M phase populations and which calculates a probability function for the S phase distribution based upon the means and standard deviations of the G1 and G2M phase (Watson *et al.*, 1987).

### Results

(*i*) *Oestrogen stimulation of T-47D cell growth, VP-16-induced DNA cleavage and cytostasis*

Figure 1a shows the growth of T-47D cells maintained in complete medium. No significant enhancement of growth occurs with oestrogen addition to the medium. In contrast, the growth rate of cells maintained in oestrogen-deprived (charcoal-stripped) medium for 2 days (Figure 1b), one week (Figure 1c) and 3 weeks (Figure 1d) declines progressively and it then becomes possible to stimulate cell growth with oestrogen.

In Figure 2, the oestrogen concentration which optimally stimulates cell growth (represented in Figure 2a as minimal doubling time) corresponds to the optimal concentration for enhancement of VP-16-induced DNA cleavage (Figure 2b) and VP-16-induced cell growth retardation (Figure 2c). These data suggest a relationship between the growth-stimulatory effect of oestrogen and the observed enhancement of VP-16-induced DNA damage.

(*ii*) *Effect of inhibitors on oestrogen-induced DNA damage enhancement*

Inhibition of the growth-stimulatory effect of oestrogen using a potent antioestrogen, 4-hydroxytamoxifen, results in antagonism of the observed enhancement of VP-16-induced DNA cleavage (Figure 3a). Simultaneous exposure of cells to oestrogen and the protein synthesis inhibitor, cycloheximide, also leads to antagonism of this effect (Figure 3b). However, inhibition of DNA synthesis using the DNA polymerase inhibitor aphidicolin does not affect the enhancement of DNA cleavage seen in oestrogen-primed cells (Figure 3c). These findings suggest that the enhancing effect of oestrogen on VP-16-induced DNA cleavage is mediated at some point by new protein synthesis but not by entry of cells into DNA synthesis.

(*iii*) *Influence of oestrogen of DNA doublestrandedness and chromatin structure*

Oestrogen stimulation of up to 72 h duration failed to cause any change in DNA scission as measured by nucleoid sedimentation (Figure 4a), and similar results were observed in cells simultaneously treated with the poly(ADP-ribosyl) transferase inhibitor 3-aminobenzamide (data not shown), suggesting that oestrogen stimulation alone may not be associated with either long-lived or transient DNA strand-break induction. DNA cleavage was also measured by alkaline unwinding in cells treated with oestrogen for 24 h, and no effect was seen over a 3 h unwinding period (Figure 4a). This implies that pre-existing DNA cleavage in samples not exposed to drug does not contribute to the observed

**Figure 2** Effect of oestrogen on cell growth, VP-16-induced DNA cleavage, and VP-16-induced cytostasis in T-47D cells: (a) Growth stimulatory effect of various oestrogen concentrations. TDx, population doubling time for oestrogen-treated cells; TDc, population doubling time for ethanol-treated control cells; (b) Effect of oestrogen concentration on percentage enhancement of DNA cleavage induced in samples exposed to 5 $\mu$M VP-16 for 1 h at 37°C when compared to ethanol control. fx, DNA cleavage in oestrogen-treated samples (see **Materials and methods**); fc, DNA cleavage in control samples; (c) Effect of oestrogen concentration on growth delay induced by 1 h exposure to 5 $\mu$M VP-16. Oestrogen-treated samples were counted in triplicate and expressed as a percentage of control values following 5 days' growth in complete medium subsequent to VP-16 treatment. In samples not exposed to VP-16, the total number of cell doublings following the 5-day incubation in complete medium consistently differed by <5% between samples initially pre-treated with either oestrogen or ethanol. Nx, number of cells in samples incubated in complete medium for five days following VP-16 exposure; Nc, number of cells in samples following 5 days' growth in complete medium without VP-16 exposure. ○, control cells; ●, oestrogen-treated cells.

enhancement of VP-16-induced DNA cleavage as assayed by alkaline DNA unwinding. That oestrogen also does not alter the overall superhelicity of DNA in T-47D cells is suggested by Figure 4b, which shows that a similar amount of ethidium bromide is required to 'untwist' negatively supercoiled DNA (thereby resulting in minimum sedimentation) in both oestrogen-treated and control cells. Chromatin accessibility to drug treatment appears to be similarly unaffected by oestrogen in either viable (Figure 4c) or freeze-thaw-permeabilised (Figure 4d) cells, since the induction of strand-breaks by either bleomycin or DNase II respectively is unchanged in cells stimulated for 24 h.

**Figure 3** Effect of inhibitors on oestrogen-induced DNA cleavage enhancement following VP-16 treatment: (a), 4-hydroxytamoxifen $10^{-7}$ M (24 h). ○, control cells; ●, oestrogen-treated cells; △, controls plus 4-hydroxytamoxifen; ▲, oestrogen-treated cells simultaneously treated with 4-hydroxytamoxifen; (b) Cycloheximide $10\,\mu g\,ml^{-1}$ (6 h). ○, control cells; ●, oestrogen-treated cells; △, control cells plus cycloheximide; ▲, oestrogen-treated (6 h) cells plus cycloheximide (6 h); (c) Aphidicolin $0.25\,\mu g\,ml^{-1}$ (24 h). ○, control cells; ●, oestrogen-treated cells; □, control cells plus aphidicolin; ■, oestrogen-treated (24 h) cells plus aphidicolin (24 h). All data points based on quadruplicate determinations.

*(iv) Time-course of events following oestrogen stimulation*

Synthesis of c-*myc* protein peaks after one hour of oestrogen stimulation, and is maintained at this level for 24 h (Figure 5a). This final level of c-*myc* protein in stimulated cells represents approximately twice that measured in control cells. The amount of DNA cleavage induced by 5 μM VP-16 also reaches double control levels in cells stimulated for 24 h, and ~50% of this increase is achieved within 8 h (Figure 5b). In contrast, no change in tritiated thymidine incorporation (Figure 5c), fraction of cells involved in DNA synthesis (Figure 5d), or rate of cell-cycle traverse (Figure 5e) is measurable after 8 h oestrogen stimulation (Figure 5c). This sequence of events suggests that oestrogen-induced mitogenesis *per se* is preceded by very early changes in protein (such as c-*myc*) synthesis, and that the observed enhancement of VP-16-induced DNA cleavage is related to an intermediate phase of cell activation which precedes stimulation of DNA synthesis.

### Discussion

The finding that oestrogen-stimulated T-47D cells sustain higher levels of VP-16-induced DNA cleavage (Figure 2b) and VP-16-induced cytostasis (Figure 2c) than do oestrogen-deprived cells extends the observation of Sullivan *et al.* (1986) that synchronously proliferating cells incur greater VP-16-induced DNA cleavage and cytotoxicity than do quiescent cells. Although not conclusive, these findings are consistent with a causative relationship between the assayed DNA cleavage and the observed toxicity of VP-16. Indirect evidence favouring this relationship has also been provided by the work of other groups interested in the mechanism of action of VP-16 (Glisson *et al.*, 1986; Estey *et al.*, 1987). This is an important point if such observations are to serve as the basis of new strategies for improving the therapeutic index of clinical cancer therapy.

The effects of cycloheximide and aphidicolin on VP-16-induced DNA cleavage in oestrogen-primed cells are in strong agreement with those reported by Chow & Ross (1987) for the effects of these inhibitors on enhanced VP-16-induced cleavage seen in synchronised cultures released from quiescence. The inhibitory effect of cycloheximide documented in both reports indicates that the enhanced drug-induced DNA cleavage witnessed in activated cell cultures is dependent at some point on new protein synthesis. This is perhaps not surprising given the very early protein synthetic events detectable immediately following stimulation (Figure 5a), events which presumably play an important role in the sequential cascade of mitogenic activation. On the other hand, the lack of antagonism induced by aphidicolin in both studies suggests that enhancement of VP-16-induced DNA cleavage occurs predominantly, if not exclusively, within activated G1-phase cells. This scenario is clearly more attractive for the design of clinical trials based on target cell stimulation than is a strategy dependent upon highly schedule-specific administration of S-phase-active drugs.

That the enhancement of VP-16-induced DNA cleavage does indeed occur in G1-phase cells is further supported by the data presented in Figure 5, which shows that VP-16-induced DNA cleavage is enhanced several hours prior to enhancement of DNA synthesis. Since c-*myc* protein levels are increased only two-fold by oestrogen stimulation of this cell system – whereas numerous studies of synchronised cell systems confirm a ten-fold increment in the expression of this gene following release of cells from quiescence (Kelly *et al.*, 1983; Dean *et al.*, 1986) – the possibility exists that only a fraction of the T-47D cell population is activated by oestrogen exposure. This hypothesis is consistent with the relatively small increase in S-phase cells documented after 24 h oestrogen exposure (Figure 5d). Furthermore, if only a subpopulation of G1-phase cells is in fact activated by oestrogen, the observed overall two-fold enhancement of VP-16-induced DNA cleavage could represent a gross underestimate of true DNA cleavage enhancement in the putative oestrogen-activated cell subset. Flow cytometric studies are now underway to clarify this issue by examining the effect of VP-16-induced DNA damage at the single-cell level.

There is now much evidence suggesting that most VP-16-induced DNA cleavage revealed by alkali- or proteinase-based DNA damage assays represents stabilised 'cleavable complexes' of DNA and the intranuclear enzyme topoisomerase II (Chen *et al.*, 1984; Ross *et al.*, 1984). The importance of chromatin structure (addressed in Figure 4) in mediating this type of DNA lesion has been highlighted by the work of Udvardy & Schedl (1986). Absolute levels of topoisomerase II have, moreover, recently been recognised to

**Figure 4** Influence of oestrogen on DNA and chromatin structure in cells not exposed to VP-16: (a) Effect of oestrogen on DNA double-strandedness as measured by either nucleoid sedimentation or alkaline DNA unwinding. Up to 72 h oestrogen exposure (●) leads to no change in nucleoid sedimentation compared to control (○). Samples pre-treated with oestrogen for 24 h (▲) show no change in the rate of alkaline DNA unwinding when compared with ethanol-treated controls (△) over a 3 h unwinding period; (b) Influence of 24 h oestrogen pretreatment on DNA superhelicity as measured by ethidium bromide titration of histone-depleted nucleoids. The amount of ethidium bromide required to induce minimum sedimentation (i.e. minimal DNA supercoiling) is not distinguishably different in oestrogen-treated (●) and control (○) samples. Datum points are based on one of two similar experiments; (c) Bleomycin-induced DNA scission as an index of chromatin accessibility in oestrogen-treated (●) and control (○) cells; (d) DNase II nicking as an index of chromatin accessibility in freeze-thaw-permeabilised cells. ○, control cells; ●, oestrogen-treated cells.

**Figure 5** Timing of detectable events following commencement of oestrogen exposure: (a) Increase in c-*myc* protein levels measured at various time-points following stimulation. Datum points are based on duplicate specimens; (b) Increase in VP-16-induced DNA cleavage following 5 $\mu$M VP-16 treatment; (c) Increase in $^3$H-thymidine incorporation during oestrogen stimulation. Standard errors are based on triplicate measurements; (d) Oestrogen-stimulated increase in rate of cell-cycle traverse. G1-phase emptying was determined for samples exposed to either oestrogen or ethanol alone for the required period by simultaneously treating samples with colcemid 60 ng ml$^{-1}$. Since this manoeuvre prevents mitotic cells re-entering G1, the rate at which cells exit from G1 can be used as an index of the rate of cell-cycle traverse. Control values at each time-point were subtracted from those of oestrogen-treated samples to yield a net percentage of G1-phase cells exiting from G1 as a consequence of oestrogen stimulation alone; (e) Effect of oestrogen stimulation on cell-cycle redistribution as measured by absolute increase in S-phase fraction relative to control. The percentage of cells in S-phase was calculated by flow cytometric analysis. ○, control cells; ●, oestrogen-treated cells.

be higher in proliferating than in quiescent cells (Heck & Earnshaw, 1986). However, the report that enzyme levels (as determined by immunoblotting) rise only in S- and G2-phase cells following stimulation – even though enhancement of VP-16-induced DNA cleavage is unaffected by inhibition of DNA synthesis using aphidicolin (Chow & Ross, 1987) – suggests that enzyme activation within G1-phase cells may accompany cellular activation, a model supported by the data presented here. Indeed, *in vitro* data have already implicated phosphorylation (Ackerman *et al.*, 1985), poly(ADP-ribosyl)ation (Darby *et al.*, 1985) and calcium-mediated pathways (Osheroff & Zechiedrich, 1987) as candidate mechanisms for topoisomerase II activation. Hence the inhibition of VP-16-induced cleavage seen with cycloheximide exposure could signify inhibited synthesis of a molecule contributing to the process of enzyme activation, rather than necessarily implicating inhibition of topoisomerase II synthesis *per se*. We are currently exploring these questions further by correlating other parameters of topoisomerase II availability with VP-16-induced DNA cleavage.

The findings presented here suggest that oestrogen stimulation of asynchronously growing human breast cancer cells is a clinically relevant strategy for improving the therapeutic index of VP-16. Since drugs more commonly used to palliate breast cancer – such as mitoxantrone – are also known to interact with topoisomerase II (Crespi *et al.*, 1986), this strategy could be rationally combined with state-of-the-art management regimens without recourse to potentially confounding cell synchronisation protocols. A clinical trial is now being designed to determine the empirical value of this approach in patients with disseminated breast cancer.

The authors would like to thank Dr John Moore for his assistance in performing the c-*myc* protein estimations, and Dr Gerard Evan for providing the c-*myc* antibody. R.J.E. was supported by the Sir Robert Menzies Memorial Trust and in part by the Royal Australasian College of Physicians.

### References

ACKERMAN, P., GLOVER, C.V.C. & OSHEROFF, N. (1985). Phosphorylation of DNA topoisomerase II by casein kinase II: Modulation of eukaryotic topoisomerase II activity *in vitro*. *Proc. Natl Acad. Sci. USA*, **82**, 3164.

AHNSTROM, G. & ERIXON, K. (1973). Radiation induced strand breakage in DNA from mammalian cells: strand separation in alkaline solution. *Int. J. Rad. Biol.*, **23**, 285.

CHEN, G.L., YANG, L., ROWE, T.C., HALLIGAN, B.D., TEWEY, K.M. & LIU, L.F. (1984). Nonintercalative antitumor drugs interfere with the breakage-reunion reaction of mammalian DNA topoisomerase II. *J. Biol. Chem.*, **259**, 13560.

CHOW, K. & ROSS, W.E. (1987). Topoisomerase-specific drug sensitivity in relation to cell cycle progression. *Mol. Cell. Biol.*, **7**, 3119.

COOK, P.R. & BRAZELL, I.A. (1975). Supercoils in human DNA. *J. Cell. Sci.*, **19**, 261.

CONTE, P.F., PRONZATO, P., RUBAGOTTI, A. *et al.* (1987). Conventional versus cytokinetic polychemotherapy with estrogenic recruitment in metastatic breast cancer: results of a randomized cooperative trial. *J. Clin. Oncol.*, **5**, 339.

CRESPI, M.D., IVANIER, S.E., GENOVESE, J. & BALDI, A. (1986). Mitoxantrone affects topoisomerase activities in human breast cancer cells. *Biochem. Biophys. Res. Comm.*, **136**, 521.

DARBY, M.K., SCHMITT, B., JONGSTRA-BILEN, J. & VOSBERG, H. (1985). Inhibition of calf thymus type II DNA topoisomerase by poly (ADP-ribosylation). *EMBO. J.*, **4**, 2129.

DAVIDSON, N.E. & LIPPMAN, M.E. (1987). Stimulation of breast cancer with estrogens: how much clinical value? *Eur. J. Cancer Clin. Oncol.*, **23**, 897.

DEAN, M., LEVINE, R.A., RAN, W., KINDY, M.S., SONENSHEIN, G.E. & CAMPISI, J. (1986). Regulation of c-myc transcription and mRNA abundance by serum growth factors and cell contact. *J. Biol. Chem.*, **261**, 9161.

EISENHAUER, E.A., BOWMAN, D.M. & PRITCHARD, K.I. (1984). Tamoxifen and conjugated estrogens followed by sequenced methotrexate and 5FU in refractory advanced breast cancer. *Cancer Treat. Rep.*, **68**, 1421.

ESTEY, E.H., SILBERMAN, L., BERAN, M., ANDERSSON, B.S. & ZWELLING, L.A. (1987). The interaction between nuclear topoisomerase II activity from human leukemia cells, exogenous DNA, and m-AMSA or VP-16 indicates the sensitivity of the cells to the drugs. *Biochem Biophys. Res. Comm.*, **144**, 787.

FARZANEH, F., ZALIN, R., BRILL, D. & SHALL, S. (1982). DNA strand breaks and ADP-ribosyl transferase activation during cell differentiation. *Nature*, **300**, 362.

FURR, B.J.A. & JORDAN, V.C. (1984). The pharmacology and clinical uses of tamoxifen. *Pharmacol. Ther.*, **25**, 127.

GAZIT, B. & CEDAR, H. (1980). Nuclease sensitivity of active chromatin. *Nucl. Acids Res.*, **8**, 5143.

GLISSON, B., GUPTA, R., SMALLWOOD-KENTRO, R. & ROSS, W. (1986). Characterisation of acquired epipodophyllotoxin resistance in a Chinese hamster ovary cell line: loss of drug-stimulated DNA cleavage activity. *Cancer Res.*, **46**, 1934.

HECK, M.M.S. & EARNSHAW, W.C. (1986). Topoisomerase II: a specific marker for cell proliferation. *J. Cell. Biol.*, **103**, 2569.

KANTER, P.M. & SCHWARTZ, H.S. (1982). A fluorescence enhancement assay for cellular DNA damage. *Mol. Pharmacol.*, **22**, 145.

KELLY, K., COCHRAN, B.H., STILES, C.D. & LEDER, P. (1983). Cell-specific regulation of the c-myc gene by lymphocyte mitogens and platelet-derived growth factor. *Cell*, **35**, 603.

KOHN, K.W., ERIKSON, L.C., EWIG, R.A.G. & FRIEDMAN, C.A. (1976). Fractionation of DNA from mammalian cells by alkaline elution. *Biochemistry*, **15**, 4628.

KUO, M. (1981). Preferential damage of active chromatin by bleomycin. *Cancer Res.*, **41**, 2439.

LATT, S.A. & STETTEN, G. (1976). Spectral studies on 33258 Hoechst and related bisbenzimidazol dyes useful for fluorescent detection of DNA synthesis. *J. Histochem Cytochem.*, **24**, 24.

LEONESSA, F., COIALBU, T. & TOAM, S. (1986). Cytotoxicity determinations in a human breast cancer cell line. *Anticancer Res.*, **6**, 1291.

LIPETZ, P.D., GALSKY, A.G. & STEPHENS, R.E. (1982). Relationship of DNA tertiary and quaternary structure to carcinogenic processes. *Adv. Cancer Res.*, **36**, 165.

LIPPMAN, M.E., CASSIDY, J., WESLEY, M. & YOUNG, R.C. (1984). A randomised attempt to increase the efficacy of cytotoxic chemotherapy in metastatic breast cancer by hormonal synchronization. *J. Clin. Oncol.*, **2**, 28.

MOORE, J.P. & EVAN, G.I. (1987). Immunoassays for oncoproteins. *Nature*, **327**, 733.

OSHEROFF, N. & ZECHIEDRICH, E.L. (1987). Calcium-promoted DNA cleavage by eukaryotic topoisomerase II: trapping the covalent enzyme-DNA complex in an active form. *Biochemistry*, **26**, 4303.

PARIDAENS, R., BLONK VAN DER WIJST, J. & JULIEN, J.P. (1985). Aminogluteithimide and estrogenic stimulation before chemotherapy for treatment of advanced breast cancer. *J. Steroid Biochem.*, **23**, 1181.

REDDEL, R.R., MURPHY, L.C. & SUTHERLAND, R.L. (1984). Factors affecting the sensitivity of T-47D human breast cancer cells to tamoxifen. *Cancer Res.*, **44**, 2398.

ROSS, W., ROWE, T., GLISSON, B., YALOWICH, J. & LIU, L. (1984). Role of topoisomerase II in mediating epipodophyllotoxin-induced DNA cleavage. *Cancer Res.*, **44**, 5857.

SMITH, P.J., ANDERSON, C.O. & WATSON, J.V. (1986). Predominant role for DNA damage in etoposide-induced cytotoxicity and cell cycle perturbation in human SV40-transformed fibroblasts. *Cancer Res.*, **46**, 5641.

SULLIVAN, D.M., GLISSON, B.S., HODGES, P.K., SMALLWOOD-KENTRO, S. & ROSS, W.E. (1986). Proliferation dependence of topoisomerase II mediated drug action. *Biochemistry*, **25**, 2248.

TAYLOR, I.W. & MILTHORPE, B.K. (1980). An evaluation of DNA fluorochromes, staining techniques, and analysis for flow cytometry. *J. Histochem. Cytochem.*, **28**, 1224.

UDVARDY, A. & SCHEDL, P. (1986). Topoisomerase II cleavage in chromatin. *J. Mol. Biol.*, **191**, 231.

WATSON, J.V., CHAMBERS, S.H. & SMITH, P.J. (1987). A pragmatic approach to the analysis of DNA histograms with a definable G1 peak. *Cytometry*, **8**, 1.

WEICHSELBAUM, R.R., HELLMAN, S., PIRO, A.S., NOVE, J.S. & LITTLE, J.B. (1978). Proliferation kinetics of a human breast cancer cell line *in vitro* following treatment with 17-beta-estradiol and 1-beta-D-arabinofuranosylcytosine. *Cancer Res.*, **38**, 2339.

ZWELLING, L.A., KERRIGAN, D. & LIPPMAN, M.E. (1983). Protein-associated intercalator-induced DNA scission is enhanced by estrogen stimulation in human breast cancer cells. *Proc. Natl Acad. Sci. USA*, **80**, 6182.